# The impact of exercise training and resveratrol supplementation on gut microbiota composition in high‐fat diet fed mice

**DOI:** 10.14814/phy2.13881

**Published:** 2018-10-28

**Authors:** Nina Brandt, Dorota Kotowska, Caroline M. Kristensen, Jesper Olesen, Ditte O. Lützhøft, Jens F. Halling, Martin Hansen, Waleed A. Al‐Soud, Lars Hansen, Pia Kiilerich, Henriette Pilegaard

**Affiliations:** ^1^ Department of Biology University of Copenhagen Copenhagen Ø Denmark; ^2^ Department of Veteranary and Animal Sciences University of Copenhagen Frederiksberg Denmark; ^3^ Department of Clinical Laboratory Sciences Faculty of Applied Medical Sciences Al‐Jouf University Qurayyat Saudi Arabia; ^4^ Department of Congenital Disorders Statens Serum Institut Artillerivej 5 DK‐2300 Copenhagen Denmark; ^5^ Present address: Danish Center for Neonatal Screening Department of Congenital Disorders Statens Serum Institute Copenhagen Denmark

**Keywords:** Exercise training, gut bacteria, high‐fat diet, microbiota, obesity, resveratrol

## Abstract

The aim of this study was to examine the effect of exercise training and dietary supplementation of resveratrol on the composition of gut microbiota and to test the hypothesis that exercise training and resveratrol can prevent high‐fat diet (HFD)‐induced changes in the gut microbiota. Mice fed a HFD supplemented with resveratrol (4 g/kg food) were protected against diet‐induced obesity, while exercise trained HFD‐fed animals (running on average 50 km/week) were not. Dietary resveratrol supplementation induced changes predominantly in the low‐abundant bacteria, while exercise training induced changes in the high‐abundant bacteria in the gut as analyzed by ADONIS test with Weighted UniFrac distances. Interestingly, the two interventions affected the gut microbiome independently of the inflammatory state of the HFD‐fed animals as assessed by the systemic serum amyloid A levels. These results suggest that both resveratrol supplementation and regular physical activity modulate the composition of murine microbiota independently of the systemic inflammatory state. Moreover, the effects of exercise training on the microbiota seem to occur without changes in adiposity, while resveratrol‐mediated alterations may relate to adipose tissue mass.

## Introduction

The gut microbiota has previously been shown to modulate lipid metabolism, energy harvest from the diet and host inflammation, potentially playing a crucial role in the development of host obesity and other metabolic disorders (Backhed et al. 2004; Greiner and Backhed 2011; Carmody and Turnbaugh 2014; David et al. 2014). Increased relative abundance of bacteria belonging to the Firmicutes phylum, and a corresponding decrease in Bacteroidetes, have been reported to be characteristic for obese mice (Ley et al. 2005; Turnbaugh et al. 2006, 2008; Cani et al. 2007). It has been hypothesized that bacteria belonging to Firmicutes phylum confer an increased capacity for energy harvest from the diet (Turnbaugh et al. 2006), which may account for some of the increase in adiposity of the host. Furthermore, an increase in the relative abundance of bacteria belonging to Firmicutes phylum has been reported to decrease endogenous production of GLP‐2 and consequently to increase LPS leakage. This has been suggested to increase host inflammation and thereby increase whole body low‐grade inflammation (Cani et al. 2007).

Resveratrol (3,5,4′‐trihydroxy‐trans‐stilbene) is a natural dietary polyphenol present in the skin of red grapes, peanuts, and berries and has been proposed to possess anti‐inflammatory, anti‐cancer, and anti‐obesity effects (Manna et al. 2000; Larrosa et al. 2009). The beneficial effects of resveratrol have been shown in several species including *Saccharomyces cerevisiae* (Howitz et al. 2003), *Caenorhabditis elegans*,* and Drosophila melanogaster* (Wood et al. 2004) as well as rodents and humans, where resveratrol has been demonstrated to protect against diet‐induced obesity and insulin resistance (Baur et al. 2006; Lagouge et al. 2006; Sung et al. 2017; Kim et al. 2018). The potential interaction between the gut microbiota and resveratrol is not yet well documented, but due to it's low bioavailability it is predicted that resveratrol reaches the colonic region of the intestine unabsorbed and unchanged, and therefore it may be subjected to enzymatic degradation by the gut microbiota (Etxeberria et al. 2015). The exact intestinal bacterial bioconversion of resveratrol is not yet known, but it has been speculated that gut bacteria may modulate the health beneficial effects of resveratrol by converting resveratrol into dihydroresveratrol, 3,4′‐dihydroxy‐trans‐stilbene and lunularin (Bode et al. 2013).

Physical activity is known to exert health‐related benefits. Exercise has been reported to share some of the same anti‐inflammatory effects as caloric restriction in treatment of obesity and diabetes (Bradley et al. 2008; Yan et al. 2012). Moreover, exercise training has been shown to reduce cell size of adipocytes, improve insulin sensitivity, and decrease the level of inflammation in adipose tissue in mice (Bradley et al. 2008; Yan et al. 2012). However, the molecular mechanism mediating these effects is not fully understood. A few studies have examined the effect of exercise training on gut microbiota, but whether exercise training directly alters gut microbiota is not known. Thus, treadmill running altered levels of cecal n‐butyrate concentration and the n‐butyrate‐producing bacteria in non‐obese rats (Matsumoto et al. 2008) and exercise training changed the gut microbiota in mice (Choi et al. 2013; Liu et al. 2017). Moreover, exercise training in mice has been reported to normalize major phylum‐level changes induced by HFD (Evans et al. 2014) and to oppose some of the obesity‐related changes in gut microbiota (Denou et al. 2016). Similar observations have been demonstrated in obese rats (Petriz et al. 2014; Welly et al. 2016). However, the impact of exercise training combined with HFD on gut microbiota and exercise training combined with HFD is still unresolved.

Therefore, the aim of this study was to investigate the impact of dietary resveratrol supplementation and voluntary exercise training on HFD‐induced changes in the gut microbiota in mice.

## Materials and Methods

### Experimental design

All mice used in this study were male C57BL/6N with loxP insertions in the *Ppargc1a* gene, and 8–10 weeks old at the initiation of the study. These mice were part of a larger study, where they served as controls for muscle‐specific PGC‐1*α* knockout mice. Hence, the use of Floxed PGC‐1*α* mice did not aim to study effects of modifications of introns in the PGC‐1*α* gene on the microbiota, but was only to take advantage of the large experimental set up. The mice were individually caged and randomly divided in to 4 different groups: (1) untrained control group receiving standard rodent chow (CON), (2) untrained group receiving HFD (HFD), (3) untrained group receiving HFD supplemented with resveratrol (HFD Res), (4) exercise trained group having access to a running wheel and receiving HFD (HFD Ex). The chow diet consisted of 20% proteins, 70% carbohydrates, and 10% fat (#1320; Altromin, Brogården, Lynge, Denmark) and the HFD consisted of 20% proteins, 20% carbohydrates, and 60% fat (#C1090‐60, containing both saturated and unsaturated fatty acids, Altromin). Pure resveratrol was kindly donated by Fluxome (Fluxome, Stenløse, Denmark) and mixed into pellets together with the HFD to a concentration of 4 g resveratrol/kg HFD as previously described (Lagouge et al. 2006). Both running distance and duration were monitored by a regular cycle computer resulting in an average 50 km/week. The mice were kept on a 12:12 h light/dark cycle and had access to water and food ad libitum. The intervention lasted for 16 weeks and body weight and food intake were monitored every second week throughout the experiment. Running wheels were blocked 24 h before the animals were euthanized by cervical dislocation at 24–26 weeks of age.

Subcutaneous (SAT) and visceral (VAT) adipose tissue were weighed and collected together with quadriceps muscle and colon stool samples, which were quickly frozen in liquid nitrogen and stored at −80°C before further processing. Moreover, trunk blood was collected and plasma was obtained by centrifugation and stored at −80°C.

Experiments were approved by the Animal Experiment Inspectorate in Denmark (#2009/561‐1689) in compliance with the European convention for the protection of vertebrate animals used for experiments and other scientific purposes (Council of Europe, no. 123, Strasbourg, France, 1985).

### Echo MRI scanning

Body composition was determined by an EchoMRI scan (EchoMRI, Echo Medical Systems, Houston, TX) 2 days before the mice were euthanized.

### RNA isolation, reverse transcription and real‐time PCR

Total RNA was isolated from crushed 15–20 mg quadriceps and 30–35 mg subcutaneous‐ and viceral adipose tissue by a modified guanidinium thiocyanate‐phenol‐chloroform extraction method (Chomczynski and Sacchi 1987; Pilegaard et al. 2000) except for the use of a TissueLyser (TissueLyser II, Qiagen, Valencia, CA) for homogenization.

Superscript II RNase H‐ system and Oligo dT (Invitrogen, Carlsbad, CA) were used to reverse transcribe the mRNA to cDNA as described previously (Pilegaard et al. 2000). Quantification of cDNA as a measure of mRNA content of a given gene was performed by real‐time PCR using an ABI 7900 sequence‐detection system (Applied Biosystems, Foster City, CA). Primers were designed from mouse‐specific database (http://www.ensembl.org/) and are presented in Table 1.

**Table 1:** Primer sequences used for real‐time PCR

	Forward Primer	Reverse Primer
TNF‐*α*	5′‐CCC TCA CAC TCA GAT CAT CTT CT‐3′	5′‐GCT ACG ACG TGG GCT ACA G‐3′
IL‐10	5′‐CAG CCA GGT GAA GAC TTT CT‐3′	5′‐GCA ACC CAA GTA ACC CTT AAA‐3′
Arg‐1	5′‐GGT GGA TGC TCA CAC TGA CA‐3	5′‐ATC ACC TTG CCA ATC CCC AG‐3′
iNOS	5′‐GCA ACC CAA GTA ACC CTT AAA‐3′	5′‐CAA ACA AGC ATA CCT GAA GG‐3′
TBP	5′‐ACC CTT CAC CAA TGA CTC CTA TG‐3′	5′‐ATG ATG ACT GCA AAT CGC‐3′

TNF‐*α*, Tumor necrosis factor alpha; IL‐10, Interleukin‐10; Arg1, Arginase‐1; iNOS, inducible Nitric Oxide Synthase; TBP, TATA‐box‐binding protein.

Real‐time PCR was performed in triplicates in a total reaction volume of 10 *μ*L using SyberGreen (Applied Biosystems). The obtained cycle threshold values reflecting the initial content of the specific transcript in the samples were converted to a relative amount using standard curves constructed from a serial dilution of a pooled sample made from all samples. TBP mRNA was unaffected by the interventions, and therefore the mRNA content of the given target gene was normalized to TBP mRNA in both quadriceps muscle and in SAT cDNA samples.

### Muscle protein

Muscle lysate was produced from ~20 mg quadriceps muscle by homogenization in ice‐cold buffer (10% glycerol, 20 mmol/L Na‐pyrophosphate, 150 nmol/L NaCl, 50 mmol/L HEPES, 1% NP‐40, 20 mmol/L *β*‐glycerophosphate, 10 mmol/L NaF, 1 mmol/L EDTA, 1 mmol/L EGTA, 20 *μ*g/mL Aprotinin, 10 *μ*g/mL Leupeptin, 2 mmol/L Na_3_VO_4_, 3 mmol/L Benzamidine, pH 7.5) for 2 min at 30 oscillations per second in a TissueLyser (TissueLyser II). The samples were set to rotate end over end for 1 h at 4°C followed by centrifugation at 17,500*g* for 20 min at 4°C. The lysates were collected as the supernatant. The protein content in the lysates was determined by the bicinchoninic acid method (Pierce Chem, Comp., IL) and lysates were prepared with sample buffer containing Sodium Dodecyl Sulfate (SDS) and boiled for 3 min at 96°C. PDH‐E1a protein content was measured by SDS‐PAGE and western blotting using self‐casted gels and a specific amount of total protein loaded. PVDF membranes were blocked in 3% fish gel, followed by incubation with primary antibodies against PDH‐E1*α* protein (1:1000, kindly provided by Professor Grahame Hardie, University of Dundee, Scotland). The membranes were incubated in HRP‐conjugated secondary antibody (Dako, Glostrup, Denmark) and protein content was visualized using LuminataTM Classico Western HRP Substrate (Millipore, Denmark). Band intensity was quantified using ImageQuant Las 4000 (GE Healthcare, Munich, Germany) and ImageQuant Imaging software. PDH‐E1a protein content was expressed in arbitrary units relative to control samples loaded on each site of each gel.

### Plasma analysis

The level of the pro‐inflammatory marker serum amyloid A (SAA) was measured in plasma samples using the Serum Amyloid A ELISA‐kit (Abcam) according to manufacturer`s instructions.

### Fecal microbiota DNA isolation and characterization

Isolation of fecal bacterial DNA from colon stool samples was performed using NM NucleoSpin Soil kit according to manufacturer's protocol. Fecal microbial composition was characterized by sequencing of the 16S rDNA V3 and V4 regions as described previously (Hansen et al., 2012). The amplified fragments with adapters and tags were quantified using Qubit™fluorometer (Invitrogen) and mixed in equimolar concentrations (4 x 10^6^ copies *μ*L^−1^) to ensure equal representation of each sample. DNA samples were sequenced in one of two‐regions of 70‐75 GS PicoTiterPlate (PTP) using a GS FLX titanium pyrosequencing system according to manufacturer's instructions (Roche) by the National High Throughput DNA Sequencing Centre, University of Copenhagen, Denmark.

### Data analyses

Data were expressed as the mean ± SE and, unless stated otherwise. Results were analyzed using ordinary one‐way analysis of variance (ANOVA) followed by Holm‐Sidak corrected multiple comparisons. For body weight, the presented statistics provide the results from multiple one‐way ANOVA tests performed over the course of the intervention. When Gaussian distribution could not be assumed, Kruskal–Wallis test was used with Dunn's post hoc test. Results were considered significant when *P* < 0.05. Values with no letters in common are significantly different (*P* < 0.05). The bar with the highest value gets the letter A, the second highest value that is significantly different from A gets the letter B, etc. Analyses were performed using GrapPpad Prism 7 software (GraphPad Software, San Diego, CA).

The analyses of 16S rDNA gene amplicon sequences were performed using the Quantitative Insights Into Microbial Ecology (QIIME, v1.6) pipeline (Caporaso et al. 2010). Cleaning the sequencing data was done using the denoise_wrapper.py script in QIIME (Reeder and Knight 2010) to remove sequencing error. Chimera removal was done by UCHIME with MicrobesOnline (May, 2013) as the reference database (Edgar et al. 2011). OTUs were picked using UCLUST (Edgar 2010) with 97% similarity and representative sequences were assigned taxonomy with the Greengenes database with 80% confidence interval. PhyloSeq (McMurdie and Holmes 2013), MetagenomeSeq (Paulson et al. 2013) and Vegan (Kronstrom et al. 2011) packages were used for further analysis in R.

## Results

### Body weight and composition

**Figure 1:**
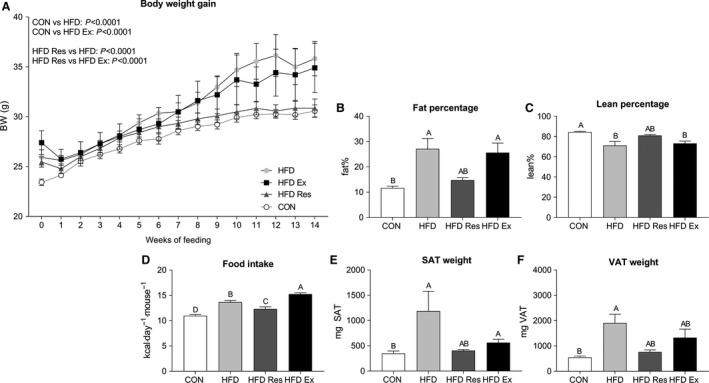
(A) Whole body weight gain curves (g) (B) fat percentage (%), (C) lean percentage (%), (D) daily food intake normalized to body weight (kcal day^−1^ mouse^−1^), (E) subcutaneous adipose tissue (SAT) weight (mg) and (F) visceral adipose tissue (VAT) weight (mg) of mice on either a chow diet (CON) or mice on a high‐fat diet (HFD), HFD supplemented with resveratrol (4 g/kg food**:** HFD Res) or HFD and exercise trained for 16 weeks. Values are mean ± SE,* n* = 8–11. Values with no letters in common are significantly different (*P* < 0.05). The bar with the highest value is marked with the letter A, the second highest value that is significantly different from A is marked with the letter B, etc.

At week 14 of the intervention, HFD feeding (HFD) increased (*P* < 0.05) total body weight of the mice compared with chow fed mice (CON). Micefed HFD supplemented with resveratrol (HFD Res) had 25% lower (*P* < 0.05) body weight than HFD mice, while the body weight of HFD Ex mice was similar to HFD‐fed mice (Fig. 1A).

Body fat percentage was twofold higher (*P* < 0.05) in both HFD and HFD Ex mice than CON mice, while there was no significant differences in the body fat percentage between HFD Res mice and any of the other groups (Fig. 1B). HFD and HFD Ex had 10% lower (*P* < 0.05) lean body mass than CON, while there was no significant difference in lean body mass between HFD Res and any of the other groups (Fig. 1C).

Food intake was higher (*P* < 0.05) in HFD, HFD Res, and HFD Ex than CON. In addition, food intake was lower (*P* < 0.05) in HFD Res than HFD and higher (*P* < 0.05) in HFD Ex than both HFD and HFD Res (Fig. 1D).

Subcutaneous adipose tissue (SAT) mass was 3‐fold and 1.5‐fold higher (*P* < 0.05) in HFD and HFD Ex mice, respectively, than CON mice, while there was no significant difference in SAT mass between HFD Res and any of the other groups (Fig. 1E).

Visceral adipose tissue (VAT) mass was 3‐fold (higher *P* < 0.05) in HFD mice than CON mice, while SAT mass in HFD Res and HFD Ex mice was not significantly different from either HFD or CON (Fig. 1F).

### Skeletal muscle oxidative marker

The protein content of the mitochondrial protein pyruvate dehydrogenase (PDH)‐E1*α* was higher (*P* < 0.05) in HFD Ex (2.03 ± 0.13) than in CON (1.33 ± 0.05), HFD (1.50 ± 0.08) and HFD Res (1.53 ± 0.08) with no difference between CON, HFD and HFD Res.

### Inflammatory markers

**Figure 2:**
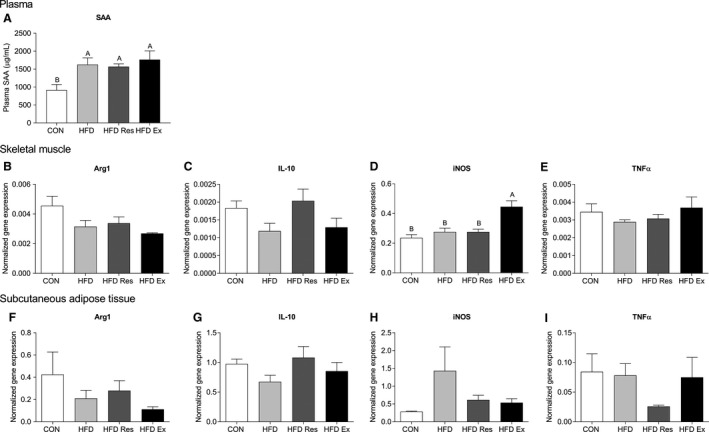
(A) ELISA measurement of plasma SAA (*μ*g/mL), (B) Arg1 mRNA, (C) IL‐10 mRNA, (D) iNOS mRNA and (E) TNF *α* mRNA levels in skeletal muscle and (F) Arg1 mRNA, (G) IL‐10 mRNA, (H) iNOS mRNA and (I) TNF *α* mRNA levels in subcutaneous adipose tissue (SAT) from mice on either a chow diet (CON) or mice on a high‐fat diet (HFD), HFD supplemented with resveratrol (4 g/kg food**:** HFD Res) or HFD and exercise trained (HFD Ex) for 16 weeks. Values are mean ± SE,* n* = 8–11. Values with no letters in common are significantly different (*P* < 0.05). The bar with the highest value is marked with the letter A, the second highest value that is significantly different from A is marked with the letter B, etc.

The marker of systemic inflammation, serum amyloid A (SAA) was higher (*P* < 0.05) in HFD, HFD Res and HFD Ex than CON with no difference between HFD groups (Fig. 2A).

There was no significant differences in the mRNA content of the pro‐ and anti‐inflammatory markers Arg1, IL‐10, and TNF*α* in skeletal muscle (Fig 2B,C and E). On the other hand, the mRNA content of the pro‐inflammatory marker iNOS was in skeletal muscle 1.7‐fold higher (*P* < 0.05) in HFD Ex than all other groups (Fig 2D).

There was no significant differences in the mRNA content of the pro‐ and anti‐inflammatory markers Arg1, IL‐10, iNOS, and TNF*α* in subcutaneous adipose tissue (Fig 2F–I).

### Microbiome composition is differently affected by exercise and resveratrol supplementation

**Figure 3:**
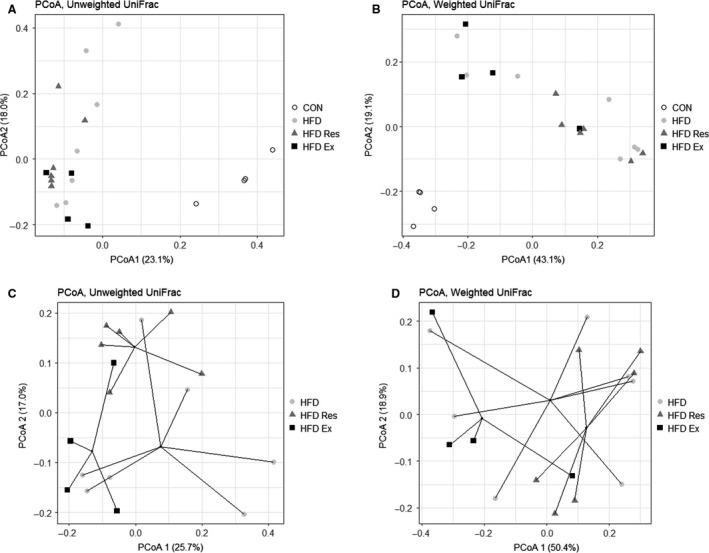
Analysis of microbiome composition in fecal samples from mice on either a chow diet (CON), a high‐fat diet (HFD), HFD supplemented with resveratrol (4 g/kg food**:** HFD Res) or HFD and exercise trained (HFD Ex) for 16 weeks using PCoA plots with (A) weighted and (B) unweighted UniFrac distances for all groups showing separation of the samples according to the high‐fat diet (HFD) (*P* = 0.001). PCoA plots for the HFD‐fed mice only (C) weighted and (D) unweighted UniFrac distances show a separation of the groups according to exercise (*P* = 0.052) and a tendency for resveratrol supplementation (*P* = 0.059), respectively. Significant differences between groups were tested using ADONIS test.

PCoA plots with Weighted and Unweighted UniFrac distances for all treatment groups showed a distinct clustering of the CON group compared with the HFD group's independent of the intervention (Fig. 3A and B). The differences were significant (*P* < 0.05) for both Weighted (high abundant bacteria) and Unweighted (low abundant bacteria), showing that the HFD affected the microbiome composition. ADONIS tests were performed to confirm the differences between groups (Table 2/Fig. 3) showing a difference between all the groups (*P* = 0.05), which most likely originated from the difference between diets (*P* = 0.05). When the HFD groups were analyzed separately, distinct centroids were observed for each treatment, suggesting a distinct microbiome composition between the three groups (Fig. 3D). This was also confirmed using ADONIS test showing a significant difference between the groups for the low‐abundant bacteria (Unweigthed UniFrac, Treatment *P* = 0.05). This difference most likely originated from the resveratrol supplementation although this intervention variable did not reach significance separately (Unweighted UniFrac = 0.059). Moreover, the impact of exercise training on the composition of the most abundant bacteria was very close to being significant (*P* = 0.052 for the Weighted UniFrac analysis).

**Table 2:** Results from ADONIS test

All groups				HFD groups			
Variable	Levels	*P*‐value, Weighted UniFrac	*P*‐value, Unweighted UniFrac	Variable	Levels	*P*‐value, Weighted UniFrac	*P*‐value, Unweighted UniFrac
Treatment	CON, HFD, HFD Res, HFD Ex	0.001	0.001	Treatment	HFD, HFD Res, HFD Ex	0.139	0.045
Diet	CON, HFD, HFD Res	0.001	0.001	Diet	HFD, HFD Res	0.124	0.059
Exercise	Sedentary, Exercise	0.192	0.167	Exercise	Sedentary, Exercise	0.052	0.121
Resveratrol	None, Resveratrol	0.055	0.024	Resveratrol	None, Resveratrol	0.158	0.059

Overview of the outcome of ADONIS tests with Weighted and Unweighted UniFracs distances for all groups or the HFD‐groups only.

**Figure 4:**
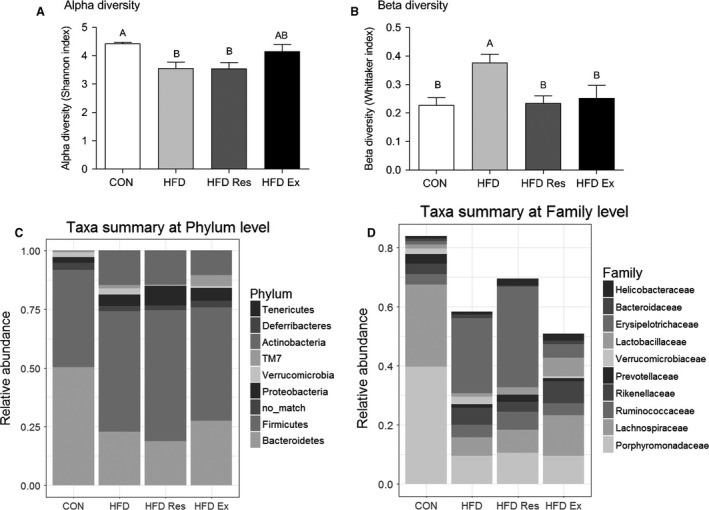
(A) Alpha diversity (Shannon index) and (B) beta diversity (Whittaker index) in fecal samples from mice on either a chow diet (CON), a high‐fat diet (HFD), HFD supplemented with resveratrol (4 g/kg food**:** HFD Res) or HFD and exercise trained (HFD Ex) for 16 weeks. Taxa summary plots at (C) Phylum level and (D) the 10 most abundant Families. *n* = 8–11. Values with no letters in common are significantly different (*P* < 0.05). The bar with the highest value is marked with the letter A, the second highest value that is significantly different from A is marked with the letter B, etc.

Alpha diversity in the fecal microbiome community was lower (*P* < 0.05) in HFD mice than CON mice (Fig. 4A) indicating a lower number of different bacterial species in HFD‐fed mice. Neither resveratrol supplementation nor exercise training affected alpha diversity compared to HFD alone. However, alpha diversity of HFD Ex mice did not differ from alpha diversity in CON mice. Beta diversity was higher in HFD than in CON mice, but returned to CON level with both resveratrol supplementation and exercise training (Fig. 4B), indicating a less uniform abundance profile in the HFD mice.

The taxa summary plot at phylum level (Fig. 4C) revealed that most of the Phyla changed in HFD relative to Control in accordance with previous studies (Turnbaugh et al. 2006, 2008; Xiao et al. 2017). In addition, the taxa summary plot at phylum level suggested changes in Bacteroidetes, Verrucomicrobia, Actinobacteria and the candidate division TM7 with exercise training relative to HFD, while resveratrol supplementation seemed to affect bacteria in the phyla of Proteobacteria and Verrucomicrobia relative to HFD. At the family level (Fig. 4D), major changes between CON and HFD were observed with a decrease in Porphyromonadaceae and Erysipelotrichaceae and an increase in Lachnospiraceae. Moreover, no changes were observed when comparing HFD Res or HFD Ex with HFD except that Erysipelotrichaceae increased and decreased in HFD Res and HFD Ex, respectively, relative to HFD.

**Figure 5:**
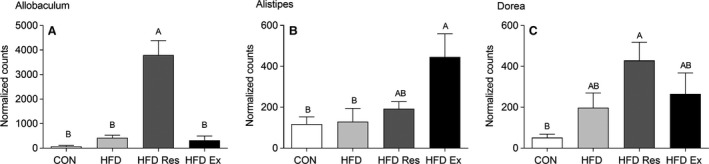
Normalized counts for (A) Allobaculum abundance (Family Erysipelotrichaceae) and (B) Alistipes abundance (Family Rikenellaceae) and (C) Dorea abundance (Family Lachnospiraceae) from mice on either a chow diet (CON) or mice on a high‐fat diet (HFD), HFD supplemented with resveratrol (4 g/kg food**:** HFD Res) or HFD and exercise trained for 16 weeks. *n* = 8–11. Values with no letters in common are significantly different (*P* < 0.05). The bar with the highest value is marked with the letter A, the second highest value that is significantly different from A is marked with the letter B, etc.

This pattern was confirmed when analyzing the normalized abundance of Allobaculum, the most abundant genus belonging to the Family of Erysipelotrichaceae. Allobaculum abundance was higher (*P* < 0.05) in HFD‐fed mice supplemented with resveratrol than all other groups (Fig. 5A). In the Rikenellaceae family, Alistipes was the most abundant genus and the abundance of Alistipes increased with exercise training, but not resveratrol supplementation, relative to HFD‐fed mice (Fig. 5B). The most abundant genus belonging to the Lachnospiraceae family was Dorea, which was higher (*P* < 0.05) in HFD Res than in CON mice (Fig. 5C).

## Discussion

This study suggests that resveratrol and exercise training, independently of obesity and systemic inflammatory status result in distinct shifts in gut microbiota composition in mice.

The present finding that resveratrol supplementation protected against diet‐induced obesity with total body weight as well as body fat content not being different from CON mice is in agreement with previous studies (Baur et al. 2006; Lagouge et al. 2006). Moreover, the present observation, that exercise training did not result in weight loss or increase in lean body mass despite the evidence for skeletal muscle mitochondrial adaptations based on the clear increase in PDH‐E1*α* protein content, is different from some, but not all previous studies examining exercise training‐induced changes in fat mass. (Bradley et al. 2008; Yan et al. 2012). However, the higher calorie intake in the HFD Ex group relative to the CON, HFD and HFD Res groups may partially explain the inconsistence with previous studies, where authors reported no difference in caloric intake between exercise‐trained and sedentary animals on control diet (Bradley et al. 2008; Yan et al. 2012).

The present observations that HFD‐fed mice changed alpha and beta diversity as well as the relative abundance of specific bacteria both at the phylum and family level are in accordance with multiple previous studies (Ley et al. 2005; Turnbaugh et al. 2006; Denou et al. 2016; Welly et al. 2016; Xiao et al. 2017). Furthermore, the finding that all three HFD groups seemed to have lower Bacteroidetes relative abundance than CON mice despite that fat percentage was higher in HFD and HFD Ex, but unchanged in HFD Res mice confirms that gut microbiota can change independently of adiposity. It may be noted that the food intake in HFD Res mice was lower than the HFD and HFD Ex, which may explain the lower adiposity. However, this does not change the observation that the HFD associated changes in microbiota was not prevented despite the lower adiposity. The present observation that *Erysipelotrichiceae* was markedly increased in HFD and HFD Res mice is in accordance with previous studies demonstrating an increase in *Erysipelotrichi* within 24 h when mice switched from chow diet to HFD (Turnbaugh et al. 2006, 2008, 2009; Hildebrandt et al. 2009; Fleissner et al. 2010; David et al. 2014). On the other hand, another study showed an increased abundance of *Erysipelotrichiceae* family and the *Allobaculum* genus within this family when mice were fed a low‐fat diet (Ravussin et al. 2012; Cox et al. 2013). Moreover, the present finding that *Erysipelotrichiceae* was not changed in HFD Ex mice compared with HFD mice is not in agreement with the previous study by Choi et al. (2013) reporting a decrease in *Erysipelotrichaceae* in exercising animals. Furthermore, the observation that Alistipes abundance was higher in HFD Ex mice than all other groups further supports an effect of exercise training on the microbiota. This finding indicates that exercise training is a stronger factor driving changes in microbiota than diet.

The observation that the abundance of *Allobaculum* was higher with resveratrol supplementation than in all other groups is in line with the previous report that the genus *Allobaculum* increased when mice were fed a low‐fat diet (Ravussin et al. 2012; Cox et al. 2013). This suggests a link between weight loss and abundance of Allobaculum, although the physiological importance of this change remains to be determined. Furthermore, the observed higher abundance of Dorea in HFD Res than CON mice supports that resveratrol supplementation influenced the microbiota. However, the present observations that resveratrol supplementation and exercise training mediated different effects on the microbiota suggest that resveratrol may not be an exercise training mimetics with regard to gut bacteria abundance and composition as previously shown for various metabolic parameters (Baur et al. 2006; Lagouge et al. 2006).

The observed changes in the gut microbiome composition in mice according to treatment in the current study were obtained using PCoA plots with Unweighted and Weighted UniFrac distances supplemented with ADONIS testing. This separation did not directly correlate with body weight or caloric intake, suggesting that obesity per se does not drive the change in microbiota composition. These findings stay in agreement with previous studies (Hildebrandt et al. 2009; Murphy et al. 2010). Hence, using RELM*β* KO mice, Hildebrandt et al. (2009) reported that it was the HFD that was responsible for the altered microbiota, rather than the obese state of the animals. A similar conclusion was reached by Murphy et al. (2010) while feeding mice both high‐fat and low‐fat diet. Furthermore, the observation that the changes in microbiota with HFD occurred without increases in the mRNA level of the pro‐inflammatory markers TNF*α* and iNOS in SAT and skeletal muscle indicates that HFD‐induced changes in gut microbiota may not be sufficient to drive alterations in tissue inflammation or alternatively that inflammation is not required for the HFD‐induced changes in gut microbiota.

On the other hand, the increase in the plasma levels of the pro‐inflammatory marker SAA with HFD in the current study suggests an enhanced systemic inflammatory state, which may have influenced the microbiota. In addition, the observed effect of exercise training on iNOS mRNA in skeletal muscle may suggest that the exercise training‐induced effects on the microbiota may be mediated via changes in the inflammatory state of skeletal muscle. However, the finding that plasma SAA was unaffected by both resveratrol supplementation and exercise training does not support that changes in the systemic inflammatory state mediated the changes in microbiota with resveratrol supplementation and exercise training in the present study.

Both resveratrol and physical activity have previously been reported to exert anti‐inflammatory effects in both rodents and human (Starkie et al. 2003; Pearson et al. 2008; Olholm et al. 2010; Woods et al. 2012), although resveratrol supplementation also has been shown to prevent an exercise training‐induced reduction in TNF*α* mRNA in human skeletal muscle (Olesen et al. 2014). The present finding that there was no effect of either resveratrol or exercise training on the inflammatory profile is therefore in contradiction with the previous studies. However, the present study determined the inflammatory markers in SAT and the quadriceps muscle, while others reported the results in VAT (Rivera et al. 2009; Kim et al. 2011) or gastrocnemius muscle (Jeong et al. 2015). In addition, the duration of treatment and dose were different between the studies and may have influenced the results. Furthermore, of notice is that the HFD in the current study did not induce inflammation in the investigated tissues, and it is therefore likely that the lack of effects of resveratrol and exercise training on the inflammatory markers is because the mice were not sufficiently affected by the HFD.

In conclusion, the present findings confirm that gut microbiota changes are independent of adiposity. Furthermore, both resveratrol supplementation and exercise training modified bacteria abundance and composition but differently.

## Conflict of Interest

None declared.

## References

[phy213881-bib-0001] Backhed, F. , H. Ding , T. Wang , L. V. Hooper , G. Y. Koh , A. Nagy , et al. 2004 The gut microbiota as an environmental factor that regulates fat storage. Proc. Natl Acad. Sci. USA 101:15718–15723. 15505215 10.1073/pnas.0407076101 PMC524219

[phy213881-bib-0002] Baur, J. A. , K. J. Pearson , N. L. Price , H. A. Jamieson , C. Lerin , A. Kalra , et al. 2006 Resveratrol improves health and survival of mice on a high‐calorie diet. Nature 444:337–342. 17086191 10.1038/nature05354 PMC4990206

[phy213881-bib-0003] Bode, L. M. , D. Bunzel , M. Huch , G. S. Cho , D. Ruhland , M. Bunzel , et al. 2013 In vivo and in vitro metabolism of trans‐resveratrol by human gut microbiota. Am. J. Clin. Nutr. 97:295–309. 23283496 10.3945/ajcn.112.049379

[phy213881-bib-0004] Bradley, R. L. , J. Y. Jeon , F. F. Liu , and E. Maratos‐Flier . 2008 Voluntary exercise improves insulin sensitivity and adipose tissue inflammation in diet‐induced obese mice. Am. J. Physiol. Endocrinol. Metab. 295:E586–E594. 18577694 10.1152/ajpendo.00309.2007 PMC2536732

[phy213881-bib-0005] Cani, P. D. , J. Amar , M. A. Iglesias , M. Poggi , C. Knauf , D. Bastelica , et al. 2007 Metabolic endotoxemia initiates obesity and insulin resistance. Diabetes 56:1761–1772. 17456850 10.2337/db06-1491

[phy213881-bib-0006] Caporaso, J. G. , J. Kuczynski , J. Stombaugh , K. Bittinger , F. D. Bushman , E. K. Costello , et al. 2010 QIIME allows analysis of high‐throughput community sequencing data. Nat. Methods 7:335–336. 20383131 10.1038/nmeth.f.303 PMC3156573

[phy213881-bib-0007] Carmody, R. N. , and P. J. Turnbaugh . 2014 Host‐microbial interactions in the metabolism of therapeutic and diet‐derived xenobiotics. J. Clin. Invest. 124:4173–4181. 25105361 10.1172/JCI72335 PMC4191041

[phy213881-bib-0008] Choi, J. J. , S. Y. Eum , E. Rampersaud , S. Daunert , M. T. Abreu , and M. Toborek . 2013 Exercise attenuates PCB‐induced changes in the mouse gut microbiome. Environ. Health Perspect. 121:725–730. 23632211 10.1289/ehp.1306534 PMC3672930

[phy213881-bib-0009] Chomczynski, P. , and N. Sacchi . 1987 Single‐step method of RNA isolation by acid guanidinium thiocyanate‐phenol‐chloroform extraction. Anal. Biochem. 162:156–159. 2440339 10.1006/abio.1987.9999

[phy213881-bib-0010] Cox, L. M. , I. Cho , S. A. Young , W. H. Anderson , B. J. Waters , S. C. Hung , et al. 2013 The nonfermentable dietary fiber hydroxypropyl methylcellulose modulates intestinal microbiota. FASEB J. 27:692–702. 23154883 10.1096/fj.12-219477 PMC3545536

[phy213881-bib-0011] David, L. A. , C. F. Maurice , R. N. Carmody , D. B. Gootenberg , J. E. Button , B. E. Wolfe , et al. 2014 Diet rapidly and reproducibly alters the human gut microbiome. Nature 505:559–563. 24336217 10.1038/nature12820 PMC3957428

[phy213881-bib-0012] Denou, E. , K. Marcinko , M. G. Surette , G. R. Steinberg , and J. D. Schertzer . 2016 High‐intensity exercise training increases the diversity and metabolic capacity of the mouse distal gut microbiota during diet‐induced obesity. Am. J. Physiol. Endocrinol. Metab. 310:E982–E993. 27117007 10.1152/ajpendo.00537.2015 PMC4935139

[phy213881-bib-0013] Edgar, R. C. 2010 Search and clustering orders of magnitude faster than BLAST. Bioinformatics 26:2460–2461. 20709691 10.1093/bioinformatics/btq461

[phy213881-bib-0014] Edgar, R. C. , B. J. Haas , J. C. Clemente , C. Quince , and R. Knight . 2011 UCHIME improves sensitivity and speed of chimera detection. Bioinformatics 27:2194–2200. 21700674 10.1093/bioinformatics/btr381 PMC3150044

[phy213881-bib-0015] Etxeberria, U. , N. Arias , N. Boque , M. T. Macarulla , M. P. Portillo , J. A. Martinez , et al. 2015 Reshaping faecal gut microbiota composition by the intake of trans‐resveratrol and quercetin in high‐fat sucrose diet‐fed rats. J. Nutr. Biochem. 26:651–660. 25762527 10.1016/j.jnutbio.2015.01.002

[phy213881-bib-0016] Evans, C. C. , K. J. LePard , J. W. Kwak , M. C. Stancukas , S. Laskowski , J. Dougherty , et al. 2014 Exercise prevents weight gain and alters the gut microbiota in a mouse model of high fat diet‐induced obesity. PLoS ONE 9:e92193. 24670791 10.1371/journal.pone.0092193 PMC3966766

[phy213881-bib-0017] Fleissner, C. K. , N. Huebel , M. M Abd El‐Bary , G. Loh , S. Klaus , and M. Blaut . 2010 Absence of intestinal microbiota does not protect mice from diet‐induced obesity. Br. J. Nutr. 104:919–929. 20441670 10.1017/S0007114510001303

[phy213881-bib-0018] Greiner, T. , and F. Backhed . 2011 Effects of the gut microbiota on obesity and glucose homeostasis. Trends Endocrinol. Metab. 22:117–123. 21353592 10.1016/j.tem.2011.01.002

[phy213881-bib-3001] Hansen, R. , R. K. Russell , C. Reiff , P. Louis , F. McIntosh , and S. H. Berry . 2012 Microbiota of de‐novo pediatric IBD: increased faecalibacterium prausnitzii and reduced bacterial diversity in crohn's but not in ulcerative colitis. Am. J. Gastroenterol. 107:1913–1922. 23044767 10.1038/ajg.2012.335

[phy213881-bib-0019] Hildebrandt, M. A. , C. Hoffmann , S. A. Sherrill‐Mix , S. A. Keilbaugh , M. Hamady , Y. Y. Chen , et al. 2009 High‐fat diet determines the composition of the murine gut microbiome independently of obesity. Gastroenterology 137:1716–1724. 19706296 10.1053/j.gastro.2009.08.042 PMC2770164

[phy213881-bib-0020] Howitz, K. T. , K. J. Bitterman , H. Y. Cohen , D. W. Lamming , S. Lavu , J. G. Wood , et al. 2003 Small molecule activators of sirtuins extend Saccharomyces cerevisiae lifespan. Nature 425:191–196. 12939617 10.1038/nature01960

[phy213881-bib-0021] Jeong, J. H. , H. G. Park , Y. R. Lee , and W. L. Lee . 2015 Moderate exercise training is more effective than resveratrol supplementation for ameliorating lipid metabolic complication in skeletal muscle of high fat diet‐induced obese mice. J. Exerc. Nutrition Biochem. 19:131–137. 10.5717/jenb.2015.15062211 PMC4523803 26244132

[phy213881-bib-3002] Kim, M. , C. Galan , A. A. Hill , W. J. Wu , H. Fehlner‐Peach , H. W. Song , et al. 2018 Critical role for the microbiota in CX3CR1+ intestinal mononuclear phagocyte regulation of intestinal T cell responses. Immunity 49:151–163. 29980437 10.1016/j.immuni.2018.05.009 PMC6051886

[phy213881-bib-0022] Kim, S. , Y. Jin , Y. Choi , and T. Park . 2011 Resveratrol exerts anti‐obesity effects via mechanisms involving down‐regulation of adipogenic and inflammatory processes in mice. Biochem. Pharmacol. 81:1343–1351. 21439945 10.1016/j.bcp.2011.03.012

[phy213881-bib-0023] Kronstrom, K. , H. Karlsson , H. Nabi , T. Oksanen , P. Salo , N. Sjosten , et al. 2011 Optimism and pessimism as predictors of work disability with a diagnosis of depression: a prospective cohort study of onset and recovery. J. Affect. Disord. 130(1–2):294–299. 21055822 10.1016/j.jad.2010.10.003

[phy213881-bib-0024] Lagouge, M. , C. Argmann , Z. Gerhart‐Hines , H. Meziane , C. Lerin , F. Daussin , et al. 2006 Resveratrol improves mitochondrial function and protects against metabolic disease by activating SIRT1 and PGC‐1alpha. Cell 127:1109–1122. 17112576 10.1016/j.cell.2006.11.013

[phy213881-bib-0025] Larrosa, M. , M. J. Yanez‐Gascon , M. V. Selma , A. Gonzalez‐Sarrias , S. Toti , J. J. Ceron , et al. 2009 Effect of a low dose of dietary resveratrol on colon microbiota, inflammation and tissue damage in a DSS‐induced colitis rat model. J. Agric. Food Chem. 57:2211–2220. 19228061 10.1021/jf803638d

[phy213881-bib-0026] Ley, R. E. , F. Backhed , P. Turnbaugh , C. A. Lozupone , R. D. Knight , and J. I. Gordon . 2005 Obesity alters gut microbial ecology. Proc. Natl Acad. Sci. USA 102:11070–11075. 16033867 10.1073/pnas.0504978102 PMC1176910

[phy213881-bib-0027] Liu, Z. , H. Y. Liu , H. Zhou , Q. Zhan , W. Lai , Q. Zeng , et al. 2017 Moderate‐intensity exercise affects gut microbiome composition and influences cardiac function in myocardial infarction mice. Front. Microbiol. 8:1687. 28919891 10.3389/fmicb.2017.01687 PMC5585143

[phy213881-bib-0028] Manna, S. K. , A. Mukhopadhyay , and B. B. Aggarwal . 2000 Resveratrol suppresses TNF‐induced activation of nuclear transcription factors NF‐kappa B, activator protein‐1, and apoptosis: potential role of reactive oxygen intermediates and lipid peroxidation. J. Immunol. 164:6509–6519. 10843709 10.4049/jimmunol.164.12.6509

[phy213881-bib-0029] Matsumoto, M. , R. Inoue , T. Tsukahara , K. Ushida , H. Chiji , N. Matsubara , et al. 2008 Voluntary running exercise alters microbiota composition and increases n‐butyrate concentration in the rat cecum. Biosci. Biotechnol. Biochem. 72:572–576. 18256465 10.1271/bbb.70474

[phy213881-bib-0030] McMurdie, P. J. , and S. Holmes . 2013 phyloseq: an R package for reproducible interactive analysis and graphics of microbiome census data. PLoS ONE 8:e61217. 23630581 10.1371/journal.pone.0061217 PMC3632530

[phy213881-bib-0031] Murphy, E. F. , P. D. Cotter , S. Healy , T. M. Marques , O. O'Sullivan , F. Fouhy , et al. 2010 Composition and energy harvesting capacity of the gut microbiota: relationship to diet, obesity and time in mouse models. Gut 59:1635–1642. 20926643 10.1136/gut.2010.215665

[phy213881-bib-0032] Olesen, J. , L. Gliemann , R. Bienso , J. Schmidt , Y. Hellsten , and H. Pilegaard . 2014 Exercise training, but not resveratrol, improves metabolic and inflammatory status in skeletal muscle of aged men. J. Physiol. 592:1873–1886. 24514907 10.1113/jphysiol.2013.270256 PMC4001758

[phy213881-bib-0033] Olholm, J. , S. K. Paulsen , K. B. Cullberg , B. Richelsen , and S. B. Pedersen . 2010 Anti‐inflammatory effect of resveratrol on adipokine expression and secretion in human adipose tissue explants. Int. J. Obes. (Lond) 34:1546–1553. 20531350 10.1038/ijo.2010.98

[phy213881-bib-0034] Paulson, J. N. , O. C. Stine , H. C. Bravo , and M. Pop . 2013 Differential abundance analysis for microbial marker‐gene surveys. Nat. Methods 10:1200–1202. 24076764 10.1038/nmeth.2658 PMC4010126

[phy213881-bib-0035] Pearson, K. J. , J. A. Baur , K. N. Lewis , L. Peshkin , N. L. Price , N. Labinskyy , et al. 2008 Resveratrol delays age‐related deterioration and mimics transcriptional aspects of dietary restriction without extending life span. Cell Metab. 8:157–168. 18599363 10.1016/j.cmet.2008.06.011 PMC2538685

[phy213881-bib-0036] Petriz, B. A. , A. P. Castro , J. A. Almeida , C. P. Gomes , G. R. Fernandes , R. H. Kruger , et al. 2014 Exercise induction of gut microbiota modifications in obese, non‐obese and hypertensive rats. BMC Genom. 15:511. 10.1186/1471-2164-15-511 PMC4082611 24952588

[phy213881-bib-0037] Pilegaard, H. , G. A. Ordway , B. Saltin , and P. D. Neufer . 2000 Transcriptional regulation of gene expression in human skeletal muscle during recovery from exercise. Am. J. Physiol. Endocrinol. Metab. 279:E806–E814. 11001762 10.1152/ajpendo.2000.279.4.E806

[phy213881-bib-0038] Ravussin, Y. , O. Koren , A. Spor , C. LeDuc , R. Gutman , J. Stombaugh , et al. 2012 Responses of gut microbiota to diet composition and weight loss in lean and obese mice. Obesity (Silver Spring) 20:738–747. 21593810 10.1038/oby.2011.111 PMC3871199

[phy213881-bib-0039] Reeder, J. , and R. Knight . 2010 Rapidly denoising pyrosequencing amplicon reads by exploiting rank‐abundance distributions. Nat. Methods 7:668–669. 20805793 10.1038/nmeth0910-668b PMC2945879

[phy213881-bib-0040] Rivera, L. , R. Moron , A. Zarzuelo , and M. Galisteo . 2009 Long‐term resveratrol administration reduces metabolic disturbances and lowers blood pressure in obese Zucker rats. Biochem. Pharmacol. 77:1053–1063. 19100718 10.1016/j.bcp.2008.11.027

[phy213881-bib-0041] Starkie, R. , S. R. Ostrowski , S. Jauffred , M. Febbraio , and B. K. Pedersen . 2003 Exercise and IL‐6 infusion inhibit endotoxin‐induced TNF‐alpha production in humans. FASEB J. 17:884–886. 12626436 10.1096/fj.02-0670fje

[phy213881-bib-3003] Sung, J. , S. Kim , J. J. T. Cabatbat , S. Jang , Y.‐S. Jin , G. Y. Jung , et al. 2017 Global metabolic interaction network of the human gut microbiota for context‐specific community‐scale analysis. Nat. Commun. 8:15393. 28585563 10.1038/ncomms15393 PMC5467172

[phy213881-bib-0042] Turnbaugh, P. J. , R. E. Ley , M. A. Mahowald , V. Magrini , E. R. Mardis , and J. I. Gordon . 2006 An obesity‐associated gut microbiome with increased capacity for energy harvest. Nature 444:1027–1031. 17183312 10.1038/nature05414

[phy213881-bib-0043] Turnbaugh, P. J. , F. Backhed , L. Fulton , and J. I. Gordon . 2008 Diet‐induced obesity is linked to marked but reversible alterations in the mouse distal gut microbiome. Cell Host Microbe 3:213–223. 18407065 10.1016/j.chom.2008.02.015 PMC3687783

[phy213881-bib-0044] Turnbaugh, P. J. , V. K. Ridaura , J. J. Faith , F. E. Rey , R. Knight , and J. I. Gordon . 2009 The effect of diet on the human gut microbiome: a metagenomic analysis in humanized gnotobiotic mice. Sci. Transl. Med. 1:6ra14. 10.1126/scitranslmed.3000322 PMC2894525 20368178

[phy213881-bib-0045] Welly, R. J. , T. W. Liu , T. M. Zidon , J. L. Rowles III , Y. M. Park , T. N. Smith , et al. 2016 Comparison of diet versus exercise on metabolic function and gut microbiota in obese rats. Med. Sci. Sports Exerc. 48:1688–1698. 27128671 10.1249/MSS.0000000000000964 PMC4987217

[phy213881-bib-0046] Wood, J. G. , B. Rogina , S. Lavu , K. Howitz , S. L. Helfand , M. Tatar , et al. 2004 Sirtuin activators mimic caloric restriction and delay ageing in metazoans. Nature 430(7000):686–689. 15254550 10.1038/nature02789

[phy213881-bib-0047] Woods, J. A. , K. R. Wilund , S. A. Martin , and B. M. Kistler . 2012 Exercise, inflammation and aging. Aging Dis. 3:130–140. 22500274 PMC3320801

[phy213881-bib-0048] Xiao, L. , S. B. Sonne , Q. Feng , N. Chen , Z. Xia , X. Li , et al. 2017 High‐fat feeding rather than obesity drives taxonomical and functional changes in the gut microbiota in mice. Microbiome 5:43. 28390422 10.1186/s40168-017-0258-6 PMC5385073

[phy213881-bib-0049] Yan, L. , L. C. DeMars , and L. K. Johnson . 2012 Long‐term voluntary running improves diet‐induced adiposity in young adult mice. Nutr. Res. 32:458–465. 22749182 10.1016/j.nutres.2012.05.006

